# Mitotic Kinases and p53 Signaling

**DOI:** 10.1155/2012/195903

**Published:** 2012-07-19

**Authors:** Geun-Hyoung Ha, Eun-Kyoung Yim Breuer

**Affiliations:** ^1^Department of Radiation Oncology, Stritch School of Medicine, Loyola University Chicago, Maywood, IL 60153, USA; ^2^Department of Molecular Pharmacology and Therapeutics, Stritch School of Medicine, Loyola University Chicago, Maywood, IL 60153, USA

## Abstract

Mitosis is tightly regulated and any errors in this process often lead to aneuploidy, genomic instability, and tumorigenesis. Deregulation of mitotic kinases is significantly associated with improper cell division and aneuploidy. Because of their importance during mitosis and the relevance to cancer, mitotic kinase signaling has been extensively studied over the past few decades and, as a result, several mitotic kinase inhibitors have been developed. Despite promising preclinical results, targeting mitotic kinases for cancer therapy faces numerous challenges, including safety and patient selection issues. Therefore, there is an urgent need to better understand the molecular mechanisms underlying mitotic kinase signaling and its interactive network. Increasing evidence suggests that tumor suppressor p53 functions at the center of the mitotic kinase signaling network. In response to mitotic spindle damage, multiple mitotic kinases phosphorylate p53 to either activate or deactivate p53-mediated signaling. p53 can also regulate the expression and function of mitotic kinases, suggesting the existence of a network of mutual regulation, which can be positive or negative, between mitotic kinases and p53 signaling. Therefore, deciphering this regulatory network will provide knowledge to overcome current limitations of targeting mitotic kinases and further improve the results of targeted therapy.

## 1. Introduction 

 Mitosis involves a highly orchestrated and fine-tuned sequence of events to properly transfer genetic information to the next generation by cell division [1, 2]. It is usually divided into five phases (prophase, prometaphase, metaphase, anaphase, and telophase) based on structure and behavior of the spindle and chromosomes, and cytokinesis begins at the end of mitosis [1, 3]. This whole process must be tightly regulated to prevent improper segregation of chromosomes [4, 5]. For this reason, cells employ a surveillance mechanism, known as the “spindle checkpoint” to ensure high fidelity of chromosome segregation in mitosis by sending a “wait signal” and thus delaying anaphase until all the chromosomes are properly aligned on the spindle apparatus (reviewed in [6]). When cells fail to delay anaphase in response to activation of spindle checkpoint, it will lead to an earlier anaphase onset, possibly causing chromosome instability, aneuploidy, and tumorigenesis [7–11]. Aneuploidy, an abnormal number of chromosomes, is a characteristic feature of cancer cells and a common cause of many genetic diseases [12, 13]. Aneuploid cells occur by an improper segregation of the chromosomes during cell division [12, 13]. The most common cause of aneuploidy is mitotic errors due to defects in “proper” mitotic kinase signaling in multiple cell cycle checkpoints, resulting in unfaithful chromosome segregation [12, 14, 15]. 

Multiple phosphorylation and proteolysis events play important roles in the regulation of mitotic progression and cytokinesis [1, 2]. Numerous proteins involved in these posttranslational events have been identified, including kinases and cysteine proteases [16–18]. One of the best understood kinases in the regulation of mitosis is cyclin-dependent kinase 1 (Cdk1) [2]. Cdks are highly conserved serine/threonine protein kinases that regulate cell cycle progression and subsequent cell division in eukaryotic cells and ubiquitously expressed throughout the cell cycle (reviewed in [19]). Among all Cdk family members, only five of them, Cdk1, Cdk2, Cdk3, Cdk4, and Cdk6, have been implicated in controlling cell cycle [20, 21]. While other Cdks are mainly involved in the early phase of cell division, Cdk1 plays a key role in several mitotic processes [2, 21, 22]. The regulation of Cdk1 has been extensively reviewed elsewhere [23–25]. Briefly, during the G2/M transition, the activation of the mitotic kinase Cdk1/Cyclin B phosphorylates a variety of substrates, such as a kinesin-related motor protein Eg5 [26], lamin [27], and condensin [28], to initiate mitotic entrance and control its progression and mitotic exit [2, 26, 27, 29]. The kinase activity appears in late G2 and peaks at metaphase [30]. At the end of the metaphase, the anaphase promoting complex (APC) (also known as cyclosome, APC/C), which is an E3 ubiquitin ligase [31], recruits cyclin B for ubiquitination and degradation to allow mitosis to proceed [32, 33]. Therefore, it is undoubtful that the perfect regulation of Cdk1/cyclin B activity is critical for normal mitotic progression. Since the discovery of Cdks, much attention has been given to the other mitotic kinases, such as Aurora kinases, Polo-like kinases (Plks), monopolar spindle 1 (Mps1), benzimidazoles 1 homolog (Bub1), and Bub1-related kinase 1 (BubR1), due to their pivotal roles in mitosis [16] as well as the relevance to cancer. Studies indicate that Aurora kinases and Plks are mainly involved in regulating the centrosome cycle and mitotic spindle formation, while Mps1, Bub1, and BubR1 regulate the spindle assembly checkpoint [34, 35]. Therefore, the tight regulation of their kinase activities is required for proper mitotic progression, which is essential for maintaining genomic integrity [5]. 

 Many studies have reported that deregulation of these mitotic kinases causes mitotic failure and aneuploidy and is closely associated with genomic instability and tumorigenesis [2, 36–38]. To defend against tumorigenesis caused by mitotic failure and guard genome stability, cells have utilized tumor suppressors, such as p53 [39] and BRCA1 [40] in a mitotic regulatory network. Because of its importance, tremendous efforts have been made to better understand the role of the functional crosstalk between mitotic kinases and tumor suppressors during mitosis. The *p53* is one of the most frequently mutated or deleted genes in human cancers and plays a role in many cellular processes, including cell growth, differentiation, senescence, and DNA repair (reviewed in [41]). In addition, p53 is a key decision maker between cell cycle arrest and apoptosis in response to DNA damage [42, 43]. The loss-of-function of p53 can trigger an increase in genome instability and cancer predisposition, suggesting that p53 is essential for the maintenance of genome stability (reviewed in [44]). The human *p53* is located on chromosome 17 (17 p13) and consists of an N-terminal transactivation domain, a central specific DNA-binding domain and a C-terminal domain, containing a tetramerization domain and regulatory region [45]. At least 20 phosphorylation sites exist in human p53 [46] and importantly, several N-terminal phosphorylation sites, such as Ser-15 [47], Thr-18 [48], and Ser-20 [49] are critical for preventing oncogenic E3 ligase MDM2-mediated p53 ubiquitination and degradation [50]. On the other hand, phosphorylation at C-terminal and a few N-terminal sites, such as Ser-362/366 [51] and Thr-55 [52] often suppresses its tumor suppressive function by destabilizing p53. These findings suggest that phosphorylation events may play significant roles in regulating p53 protein stability and function. 

**Figure 1:**
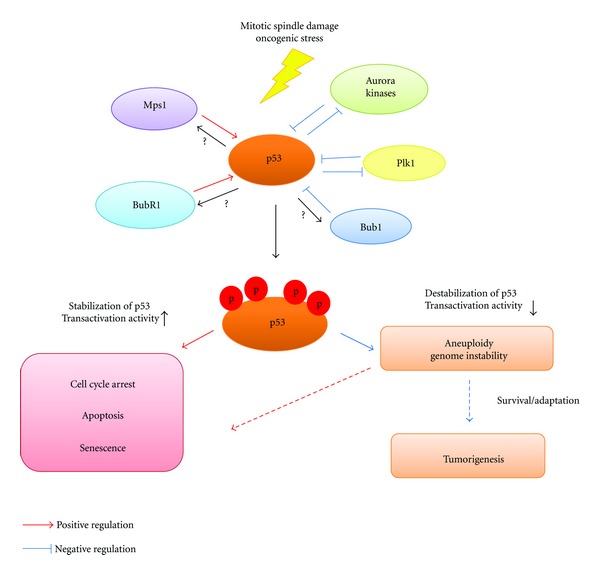
A model for regulatory networks of mitotic kinases controlling p53 signaling.

 Under normal circumstances, cells induce the p53-dependent transcriptional activation, cell cycle arrest, and apoptosis in response to mitotic defects or DNA damage [53, 54]. However, cells lacking functional p53 due to deregulation of mitotic kinases, such as Aurora A [55], Plk1 [56], and Bub1 [57], do not undergo these cellular events and thus lead to genome instability, resulting in aneuploidy [15]. Phosphorylation of p53 by Mps1 [58] and BubR1 [59] stabilizes p53 and appears to antagonize the function of Aurora A, Plk1, and Bub1 in p53 signaling. Studies have shown that p53 can also regulate the expression and function of these kinases [60–64], suggesting that there may be mutual regulatory interactions between mitotic kinases and p53 in a mitotic signaling network (Figure 1).

 In this paper, we will specifically focus on the classic mitotic kinases, including Aurora kinases, Plks, Bub1, Mps1, and BubR1, and their roles in regulating p53 protein stability and activity.

## 2. Negative Regulation of p53 

### 2.1. Aurora Kinases

Aurora kinases belong to a highly conserved family of serine/threonine kinases crucial for chromosome segregation, condensation, and spindle assembly [1]. The first Aurora kinase was discovered in *Drosophila melanogaster* mutants having defects in mitotic spindle-pole formation [65]. Subsequently, homologues of Aurora kinases have been identified in various species. In budding yeast, there is a single Aurora kinase, known as increase-in-ploidy 1 (Ipl1) [66]. The *Ipl1* gene is essential for maintaining genome stability through its roles in chromosome segregation, spindle checkpoint, mitotic spindle disassembly, and cytokinesis [67, 68]. *Caenorhabditis elegans* has two Aurora kinases, Aurora/Ipl1-related-1 and -2 (AIR-1 and AIR-2), and they are thought to be key regulators of mitotic spindle assembly and dynamics [69, 70]. Three members of Aurora kinase family, Aurora A, B, and C, have been identified in mammalian cells [1]. The Aurora kinase family share a highly conserved C-terminal catalytic domain and a short N-terminal domain [71], and function in the regulation of mitosis and cytokinesis [72]. Deregulation of Aurora kinases causes a defect in spindle assembly, checkpoint function, and cell division, leading to chromosome missegregation or polyploidization [73]. Not surprisingly, overexpression of Aurora kinases is often found in a variety of human cancers [74–76]. Since the discovery of Aurora kinases, many efforts have been made to improve our understanding of their biological and physiological function in mitosis and the regulatory mechanisms relevant to cancer.

 Aurora A is ubiquitously expressed in proliferating cells and its activity is tightly regulated through the cell cycle [77]. Both the expression level and kinase activity of Aurora A are significantly increased from the late G2 through the M phase [74, 78] and become low during interphase [79]. Aurora A plays a key role in mitotic spindle formation, centrosome maturation [80], and activation of cell cycle regulators, such as Plk1 [81, 82] and Cdk1 [83]. Deregulated expression and activity of Aurora A can generate aneuploidy phenotype due to centrosome amplification and spindle multipolarity [84]. Numerous substrates of Aurora A have been identified, including p53 [85], human enhancer of filamentation 1 (HEF1) [86], TPX2 [87], Ajuba [88], Plk1 [81], BRCA1 [89], and transforming acidic coiled-coil 3 (TACC3) [90]. Human p53 is directly phosphorylated by Aurora A at two sites, Ser-215 [85] and Ser-315 [55], *in vitro* and *in vivo*. Phosphorylation of Ser-215 but not Ser-315 inhibits p53 DNA binding and its transactivational activity [85], whereas phosphorylation of Ser-315 induces MDM2-mediated p53 ubiquitination and subsequent degradation [55]. These findings suggest that Aurora A-mediated phosphorylation of p53 plays a negative regulatory role in p53 protein stability and its downstream signaling pathways. In response to DNA damage, p53 interacts with the heterogeneous nuclear ribonucleoprotein K (hnRNPK), a transcriptional coactivator of p53, and induces the p53 signaling pathway [91]. hnRNPK is phosphorylated on Ser-379 by Aurora A and this phosphorylation disrupts its interaction with p53 [92], suggesting that Aurora A can indirectly/negatively regulate p53 function via hnRNPK phosphorylation. Interestingly, a recent study shows that Aurora A can positively regulate p53 protein expression levels and *vice versa* [60]. In addition,* Xenopus* p53 can block *Xenopus* Aurora A's ability to transform cells [61], further supporting the existence of crosstalk between Aurora A and p53. 

 Aurora B is a member of the chromosome passenger complex (CPC), a key regulator of chromosome segregation, histone modification, and cytokinesis during mitosis [93, 94]. The CPC is composed of Aurora B and its nonenzymatic regulatory subunits inner centromere protein (INCENP), Borealin and Survivin [94], required for the activity, localization, and stability of Aurora B [93]. Aurora B governs the spindle assembly checkpoint and manages the correct chromosome segregation and cytokinesis during mitosis [72, 95]. Inhibition of Aurora B results in a failure of mitosis due to defects in chromosome segregation and microtubule dynamics [96], leading to endoreduplication and further polyploidization [97, 98]. Aurora B phosphorylates p53 on Ser-183, Ser-269, and Thr-284, all located within the p53 DNA binding domain; however, phosphorylation on these sites does not lead to degradation of p53, instead, phosphorylation on Ser-269 and Thr-284 inhibits its transcriptional activity [46]. These findings suggest that the hyperactivation or overexpression of Aurora A and B may compromise p53's tumor suppressive function via its destabilization and inactivation. 

 In contrast to Aurora A and B, the biological function of Aurora C has not been well-defined. Aurora C was first discovered in mouse sperm and eggs using a kinase screen [99]. While Aurora A and B are ubiquitously expressed in many different tissues and cells, especially actively dividing cells [98, 100, 101], Aurora C is predominantly expressed in the testis [99, 102], but not in other normal mouse somatic tissues and cell lines and mitotic spermatogonia [103]. In addition, its loss-of-function leads to a failure of meiosis [103, 104]_,_ indicating that Aurora C plays a critical role in meiosis. Recent studies show that Aurora B and C have similar structural and functional properties [105]. Inhibition of Aurora C causes aneuploidy, just like Aurora B, and furthermore, simultaneous inhibition of Aurora B and C causes a higher frequency of aneuploidy [105]. Aurora C can also support mitotic progression in the absence of Aurora B [105]. Moreover, overexpression of Aurora C causes abnormal cell division due to amplified centrosomes and micronucleation [101, 106],   suggesting that Aurora C may be involved in mitosis as well. Unlike Aurora A and B, the role of Aurora C in the regulation of p53 protein stability and function has not been reported yet. 

### 2.2. Polo-Like Kinase 1 (Plk 1)

Plks are a family of highly conserved serine/threonine protein kinases [107] named after the *polo* gene of *Drosophila melanogaster, *whose mutation causes a high frequency of abnormal mitosis and meiosis [108]. Subsequently, its homologues have been found in other species, including Cdc5 in *Saccharomyces cerevisiae,* [109], Plo1p in *Schizosaccharomyces pombe* [110], Plc1, Plc2, and Plc3 in *Caenorhabditis elegans* [111, 112], and Plx1, Plx2, and Plx3 in *Xenopus laevis *[113–115]. In mammals, five Plks have been identified: Plk1 (also known as serine/threonine-protein kinase 13, STPK13), Plk2 (also known as serum-inducible kinase, SNK), Plk3 (also known as fibroblast-growth-factor-inducible kinase, FNK; proliferation-related kinase, PRK; or cytokine-inducible kinase, CNK), Plk4 (also known as SNK akin kinase, SAK or serine/threonine-protein kinase 18, STK18), and Plk5 [116–125]. All Plks are abundantly expressed in tissues exhibiting high levels of mitotic activity [120] and share two conserved domains, an N-terminal Ser/Thr kinase domain and a C-terminal polo-box domain (PBD) [107, 126].

 It is now widely recognized that Plks are key regulators of mitosis, meiosis, and cytokinesis [107, 127, 128] as well as DNA damage response [107, 123, 126]. Deregulation of Plks leads to centrosome abnormalities, aneuploidy, and genomic instability [129], possibly leading to cancer development [130]. This may explain why deregulated expression of Plks is often detected in many types of cancer (reviewed in [37]).

 Plk1 reaches peak expression during G2/M phase and kinase activity during mitosis [128, 129]. Plk1 is the best characterized family member among others and plays an essential role in centrosome maturation and separation [131], spindle assembly and formation [110], G2 checkpoint recovery through activating cyclin-dependent kinase [132], mitotic exit [113], and cytokinesis [133]. Studies have shown that cancer cells display a higher dependency on Plk1 for cell proliferation and mitosis [134, 135] than primary cells [136]. Deregulated expression and activity of Plk1 generate abnormal centrosomes [129] and initiate malignant transformation [137]. Not surprisingly, deregulation of Plk1 is often found in many types of cancer, including melanoma [138, 139], lung [140], head and neck [141, 142], breast [143], and ovarian cancer [144] with poor prognosis. Mounting evidence suggests that Plk1 negatively regulates p53 through direct and indirect mechanisms [145]. p53 is phosphorylated by Plk1 *in vitro *and its transcriptional activity and proapoptotic function are inhibited by direct interaction and phosphorylation of Plk1 [146]. Plk1 can also inhibit p53 phosphorylation at Ser-15, which is required for blocking p53-MDM2 interaction, thereby facilitating p53's degradation [56]. Plk1 phosphorylates topoisomerase I-binding protein (Topors) at Ser-718 [145]. Topors is a p53 and topoisomerase I binding protein [147]and functions as both ubiquitin and SUMO-1 E3 ligase for p53 [148, 149]. Phosphorylation of Topors on Ser-718 by Plk1 inhibits sumoylation of p53, whereas ubiquitination and subsequent degradation of p53 is enhanced, thereby suppressing p53 function [145]. G2 and S-phase-expressed 1 (GTSE1) is critical for G2 checkpoint recovery [150, 151] and negatively regulates transactivational and apoptotic activity of p53 [150, 152]. Phosphorylation of GTSE1 on Ser-435 by Plk1 promotes its nuclear localization and subsequently, shuttles p53 out from the nucleus to the cytoplasm [151, 152], leading to p53 degradation and inactivation during G2 checkpoint recovery [151]. Plk1, p53, and Cdc25C have shown to form a complex [56, 153]. Plk1 phosphorylates Cdc25C on Ser-198 [132, 154] and presumably, this phosphorylation may contribute to p53 destabilization [56, 153]. Interestingly, there is evidence that p53 can serve as a negative regulator of Plk1 by binding to the promoter of Plk1 and thus inhibiting its activity [62, 63].

 The *Plk2* and *Plk3* are serum-inducible immediate early response genes [155] and activated near the G1/S phase transition [118, 156]. Evidence suggested that both *Plk2* and *Plk3* function as tumor suppressors in the p53-mediated signaling pathways to protect cell from DNA damage or oxidative stress (reviewed in [157]). Activation of Plk2 is required for centrosome duplication [156] and may have an important role in replication stress checkpoint signaling through the interaction with Chk1, Chk2, and p53 [158]. Plk2 appears to be a transcriptional target of p53 and its expression is induced after DNA damage in a p53-dependent manner [159]. Promoter analysis has shown the possible existence of p53 binding homology element (p53RE) in the basal promoter of *Plk2* and furthermore, *Plk2* is transcriptionally regulated by p53RE in human thyroid cells [160].

 Plk3 plays an important role in the regulation of mitosis and DNA damage checkpoint [161, 162]. Its kinase activity peaks during late S and G2 phase [116]. The gene expression signature of* Plk3* has shown deregulated expression of *Plk3* in various types of cancers [122, 163], such as head and neck squamous cell carcinomas [164] and colon cancer [165]. Overexpression of Plk3 suppresses cell proliferation [166] and induces chromosome condensation [167]. In response to DNA damage, Plk3 is activated in an ATM-dependent manner [162] and subsequently, mediates ATM-dependent Chk2 phosphorylation and activation [161, 162]. Plk3 also inhibits entry into mitosis by phosphorylating Cdc25C [168, 169] and induces p53-dependent apoptosis [169]. In addition, Plk3 interacts with and phosphorylates p53 at Ser-20 [169], thereby preventing the interaction between p53 and MDM2, with the effect of stabilizing p53. 

 Plk4 shares relatively little sequence homology with other members of Plks [170]. Plk4 is essential for centrosome duplication [171, 172] and mouse embryonic development [173]. Its protein expression peaks during mitosis [174]. The loss-of-function of Plk4 causes a failure of cell division, possibly leading to aneuploidy and polyploidy, which may in turn contribute to tumorigenesis [171]. Plk4 interacts with proteins involved in the cellular response to DNA damage, such as p53 [175], Cdc25C [176], and Chk2 [177], suggesting that Plk4 may play an important role in the DNA damage response signaling [178]. Plk4 also binds to and phosphorylates p53 [173, 175, 178], possibly affecting protein stability and activity of p53 [178], although phosphorylation site(s) are currently unknown. Overexpression of Plk4 promotes centriole overduplication [172] and is found in human colon cancer [179].

A fifth member of the Plk family, Plk5, is mainly expressed in differentiated tissues, such as the brain, eye, and ovary [180], whereas it is undetectable in proliferating tissues [181]. Plk5 is involved in the process of neurite formation [181] and DNA damage response [123], rather than mitotic process. Nucleotide sequence analysis of *Plk5* shows that the promoter region of *Plk5* contains several p53 binding motifs; however, no such regulatory mechanisms have yet been found [123]. Interestingly, recent studies demonstrated that Plk5 is significantly downregulated by promoter hypermethylation in human brain tumors and its overexpression suppresses cell proliferation and malignant transformation by *Ras* oncogene, suggesting that *Plk5* may function as a tumor suppressor gene in brain cancer [123, 181]. 

### 2.3. Budding Uninhibited by Benzimidazoles 1 Homolog (Bub1)

 Bub1 belongs to a small group of serine/threonine kinases that play multiple roles in chromosome segregation and spindle checkpoint during mitosis [182]. Bub1 was originally identified in genetic screens of *Saccharomyces cerevisiae *along with mitotic arrest-deficient 1, 2, and 3 (Mad1, Mad2, and Mad3 (BubR1) in mammals), Bub3, and Mps1 [183, 184]. All of these proteins play critical roles in the mitotic checkpoint signaling [183, 184]. Deregulated Bub1 expression and its kinase activity have been associated with chromosomal instability, aneuploidy, and several forms of human cancer [185–187]. APC/C is involved in controlling sister chromatid separation and mitotic exit [188]. Bub1 ensures that activation of APC/C is delayed until all the chromosomes have achieved proper bipolar connections to the mitotic spindle, by phosphorylating Cdc20, a key regulator of APC/C activity [189]. Phosphorylation of H2A on Ser-121 by Bub1 in fission yeast prevents chromosome instability via maintenance and localization of Sgo1 (Shugoshin), a protector of centromeric cohesion [190–192]. Bub1 interacts with p53 at kinetochores in response to mitotic spindle damage and negatively regulates p53-mediated cell death [57]. It has shown that SV40 large T antigen (LT) phosphorylates p53 on Ser-37 in a Bub1-binding manner [193]. In addition, purified Bub1 directly phosphorylates p53 on Ser-37 *in vitro*, possibly inducing cellular senescence [193]. An interesting observation has been reported that the loss of both Bub1 and p53 causes a failure in p53-mediated cell death signaling, thereby leading to the accumulation of cells with aneuploidy and polyploidy [194]. 

## 3. Positive Regulation of p53 Activation 

### 3.1. Monopolar Spindle 1 (Mps1)

Mps1 has an essential role in centrosome duplication, checkpoint signaling, cytokinesis, and development in organisms from yeast to mammalian [195–197]. Kinases structurally related to human Mps1 were identified in various organisms, including Mph1p in *Schizosaccharomyces pombe *[198], PPK1 in* Arabidopsis thaliana *[199], xMps1in* Xenopus laevis *[200] and mMps1 in mouse [201]. Mps1 acts as a dual-specificity protein kinase that can phosphorylate serine/threonine as well as tyrosine residues [198, 202] and is highly expressed during mitosis [203]. Deregulation of Mps1 causes a high frequency of chromosome missegregation and aneuploidy [203, 204] and fails to induce apoptosis in response to spindle damage [196]. The kinase activity of Mps1 is critical for maintaining chromosome stability by phosphorylating other protein substrates [205, 206]. For instance, Mps1 is crucial for Aurora B activity and chromosome alignment by phosphorylating Borealin/Dasra B, a member of CPC that regulates Aurora B [205]. In addition, Mps1 phosphorylates Blm, which is a bloom syndrome product and a member of the RecQ helicases [207], at Ser-144 [206]. Blm phosphorylation by Mps1 is important for the faithful chromosome segregation [206]. Mps1 phosphorylates p53 at Thr-18, and this phosphorylation is critical for the stabilization of p53 by interfering with MDM2 binding [58]. Mps1-mediated p53 phosphorylation is also required for the activation of p53-dependent postmitotic checkpoint [58]; thus, inhibition of Mps1 kinase activity causes a defective postmitotic checkpoint and chromosome instability [58, 208]. These findings suggest that Mps1-mediated phosphorylation and subsequent stabilization of p53 may play an important role in the activation of p53 after spindle damage as well as the prevention of aneuploidy/polyploidy [58, 208]. Interestingly, a recent study shows that increased expression of Mps1 is associated with an increased *p53* mutation, a basal-like phenotype of breast cancer and a poor prognosis outcome [209]. These findings suggest that both the expression and function of Mps1 and p53 are highly correlated and critical for effective and faithful mitosis to maintain genome stability.

### 3.2. Bub1-Related Kinase 1 (BubR1)

BubR1 is the mammalian homolog of yeast Mad3 and Bub1 [185, 210]. It has shown to play an essential role in mitotic checkpoint activation and subsequent apoptotic events to prevent the adaptation of abnormal and unstable mitotic cells with chromosome instability [59, 211]. During mitotic checkpoint activation, BubR1 directly binds to APC/C and Cdc20 and subsequently, inhibits the E3 ligase activity of APC/C by blocking the binding of Cdc20 to APC [212], suggesting that BubR1 plays an essential role in stabilization of kinetochores-microtubule attachment [213]. Several studies have shown that BubR1 deficiency causes a loss of checkpoint control, abnormal mitosis, genomic instability, and tumorigenesis as well as a compromised response to DNA damage [214]. For instance, mice with *BubR1* haploinsufficiency display a genetic instability phenotype due to underlying defects in DNA repair and chromosomal segregation [215]. Moreover, the complete loss of BubR1 leads to early embryonic lethality [216]. The reduced protein level of BubR1 promotes cellular senescence in mouse embryonic fibroblasts [217]. Increasing evidence suggests that a positive regulatory loop between p53 and BubR1 exits [218]. BubR1 interacts with and phosphorylates p53, thereby stabilizing p53 in response to spindle damage [59]. The expression level of p53 protein is reduced in BubR1-deficient cells, possibly leading to malignant transformation [214]. In *p53*-null cells, inhibition of BubR1 expression enhances chromosomal instability and polyploidy; conversely, overexpression of BubR1 restores the checkpoint function, suppresses centrosome amplification, and selectively eliminates cells with amplified centrosomes [64]. Interestingly, BubR1 transcription and expression are largely controlled by p53 [64].Despite of its important function, mutations of *BubR1* in cancers are very rare [1, 219].

## 4. Conclusions 

Thanks to advances in proteomics technology, many of the substrates for mitotic kinases have been identified, such as those listed above; however, the functional significance of these phosphorylation events has not been explored thoroughly. Therefore, dissecting the functional consequences of mitotic kinase-mediated phosphorylation should be given high priority to better understand their roles in mitosis. 

 It appears that there is a very well-organized interactive feedback loop between p53 and mitotic kinases in cell cycle progression. p53 tightly and negatively regulates the expression and activity of mitotic kinases, such as Aurora A, Plk1, and Bub1, thereby inhibiting cell proliferation and survival signaling in normal mitosis [61–64]. Protein stability and transcriptional and apoptotic activity of p53 can be also negatively regulated by mitotic kinases-mediated phosphorylation of p53 (summarized in Table 1) [55, 56, 85, 146]. On the other hand, Mps1 and BubR1 are thought to be positive regulators of p53 and may have an important role in antagonizing the function of Aurora kinases, Plk1, and Bub1 in the regulation of p53 signaling during mitosis [209, 220]. When this critical feedback loop is disrupted (e.g., by mutation of *p53 * or deregulation of mitotic kinases), p53 cannot be activated when damage occurs to the mitotic spindle, thereby inducing mitotic slippage and preventing apoptosis (Figure 1) [221, 222]. Based on these studies, we speculate that the status of both mitotic kinases and p53 may be critical for cell fate decisions in mitotic cells.

 Despite promising preclinical data of targeting mitotic kinases for cancer therapy, many challenges still remain to be overcome, such as safety issues and selection of patient population. Studies have demonstrated that current mitotic inhibitors that target mitotic kinases have major side effects because mitotic kinases are mainly expressed in actively proliferating cells (both normal dividing cells and cancer cells) [2]. Therefore, selecting the right drugs and doses for right patients may be the key to successful cancer therapy. 

 Studies have shown that depletion/inhibition of Aurora A, Aurora B, Plk1, or Bub1 induces cellular senescence or cell death in a p53-dependent or -independent but p73-dependent manner in many different cell types [217, 223–231]. Importantly, p53-deficient/mutated cells are more sensitive to inhibitors targeting Aurora kinases or Plk1 than cells with wild-type p53, due to a significant increase in cellular senescence and cell death [227, 231, 232], suggesting that patients with p53 deficiency and mutations may benefit from inhibitors targeting Aurora kinases, Plk1, or Bub1. Mps1 and BubR1-mediated p53 phosphorylation are required for p53 activation to properly induce cell death in a p53-dependent manner in response to mitotic spindle damage [58, 59, 209]. Inhibition of Mps1 or BubR1 appears to be disabling a p53-mediated cell death signaling pathway, possibly leading to accumulation of aneuploid/polyploid cells in response to mitotic spindle damage or oncogene-induced DNA damage [59, 217]. Moreover, a recent study shows that depletion/inhibition of Mps1 fails to kill p53-deficient/mutated cells more efficiently than cells expressing wild-type p53 [233], suggesting that Mps1 or BubR1 inhibition may offer a better therapeutic benefit for cancer patients expressing wild-type p53. These finding suggest that the status of p53 is a very attractive maker capable of selecting patients who will benefit from these mitotic kinase inhibitors.

## Figures and Tables

**Table 1:** Mitotic kinases-mediated p53 phosphorylation and the possible consequences.

Mitotic kinases	Phosphorylation sites	Outcome	References
Aurora kinases			
Aurora A	Ser-215	Inhibition of DNA binding and transcriptional activity	[85]
	Ser-315	Protein destabilization	[55]
Aurora B	Ser-183	Unknown	[46]
	Ser-269/Thr284	Inhibition of transcriptional activity	
Aurora C		Unknown	

Polo-like kinases			
Plk1	Unknown	Inhibition of transcriptional and proapoptotic activity	[146]
Plk2		Unknown	
Plk3	Ser-20	Protein stabilization	[169]
Plk4	Unknown	Possibly affecting protein stabilization and transcriptional activation	[178]
Plk5		Unknown	

SAC kinases			
Bub1	Ser-37	Possibly inducing cellular senescence	[193]
Mps1	Thr-18	p53 stabilization	[58]
		p53-dependent postmitotic checkpoint activation	
BubR1	Unknown	p53 stabilization	[59]
